# Different intensity extension methods and their impact on entrance dose in breast radiotherapy: A study

**DOI:** 10.4103/0971-6203.56079

**Published:** 2009

**Authors:** A. Sankar, J. Velmurugan

**Affiliations:** 1 Department of Radiation Therapy, Salmaniya Medical Complex, Salmaniya, Kingdom of Bahrain; 2 Department of Physics, Anna University, Chennai, India; * At present working in Liverpool Hospital, Liverpool, Sydney, Australia

**Keywords:** IMRT, virtual bolus and skin flash

## Abstract

In breast radiotherapy, skin flashing of treatment fields is important to account for intrafraction movements and setup errors. This study compares the two different intensity extension methods, namely, Virtual Bolus method and skin flash tool method, to provide skin flashing in intensity modulated treatment fields. The impact of these two different intensity extension methods on skin dose was studied by measuring the entrance dose of the treatment fields using semiconductor diode detectors. We found no significant difference in entrance dose due to different methods used for intensity extension. However, in the skin flash tool method, selection of appropriate parameters is important to get optimum fluence extension.

## Introduction

Dose heterogeneity is an important factor in the cosmesis of radiation therapy for breast cancer patients. Dose heterogeneity within treatment volume is high, up to 15%, in conventional radiotherapy techniques like tangential wedge pairs. This is due to contour variation of the breast and the presence of low density lung structure in treatment volume.[1–2] This heterogeneity is also high, up to 20%, in patients with large breasts.[3] Many studies have shown that dose homogeneity within the breast can be significantly improved with the help of Intensity Modulated Radiation Therapy (IMRT).The use of IMRT not only improves dose homogeneity but also helps minimize the dose to critical structures like heart and ipsilateral lung present in the treatment volume.[4–6] However, during breast IMRT planning, as with other sites, intrafraction motion of organ and interfraction set up errors need to be accounted in the treatment planning process. This results in the extension of the breast Planning Target Volume (PTV) outside the skin region. Most of the commercially available Treatment Planning Systems (TPS) assign a zero dose region outside the skin. This leads to the failure in IMRT optimization as the iterative process continuously increases intensity outside the skin to increase the dose in this region to prescription level. One solution to this problem is to add artificial bolus in the region of PTV outside the skin during optimization, as suggested in ICRU 62.[7] This is known as the virtual bolus method. In some planning systems it is possible to extend the fluence of the optimized beam outside the skin using a special tool known as skin flash tool. In this method, optimization is performed with the PTV not extending beyond the skin. After this the optimized beam intensity is extended outside the skin using skin flash tool to account for the intrafraction motion and interfraction setup errors.

This work studies the effect of different planning techniques on skin dose in breast radiotherapy. The plans were generated on a hypothetical target volume and Organ at Risk (OAR) drawn on phantom images. We also study the effect of intensity extension produced by two different methods on skin dose in IMRT. The dose measurements were performed using diode detectors, and the skin dose is obtained by measuring the entrance dose at the measurement point due to different beams.

## Materials and Methods

### Planning and Delivery System

Eclipse (Version 8.0) treatment planning system is used to generate treatment plans. A Clinac 600CD series (Varian) linear accelerator with millennium Multi Leaf Collimator (MLC) is used to deliver the treatment plans. The millennium MLC present in the accelerator is a tertiary collimating system and consists of 60 pairs of single focused MLC leaves with two different leaf widths projected at the isocenter plane. Of the 60 pairs of leaves the middle 40 pairs project a leaf width of 5mm at isocenter. The outer 20 pairs project a width of 10 mm at isocenter. All plans are generated using 6 MV photon beams and the IMRT plans are delivered by the dynamic method.

### Study Case

**Figure 1:**
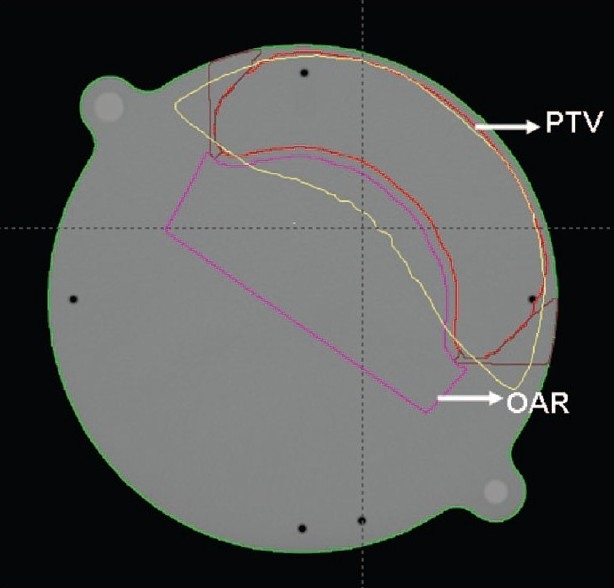
A hypothetical target volume and OAR drawn on a phantom image set for the study

A hypothetical target volume which resembles the shape of a breast in the axial plane is drawn on a PTW cylindrical phantom CT data set. This is used to generate all the plans in our study. A dose limiting structure (OAR) is also drawn adjacent to the target volume to restrict the prescription dose to target volume [figure 1]. A prescription dose of 100 cGy to target volume has been used in all plans.

### PTV Margin

Intrafraction motion of breast due to respiration and interfraction setup errors in patient positioning requires a sufficient CTV to PTV margin in the breast treatment. Studies have shown that intrafraction motion of breast in supine patient positioning is maximum 2mm in normal breathing and 5mm in deep breathing.[8–9] The interfraction setup errors vary up to two cm based on individual patients.[9–10] Though the intrafraction motion can be managed with the help of breath hold or gating techniques the interfraction setup error dominates the Cumulative Maximum Movement Error (CMME) of patient positioning and still requires significant CTV to PTV margin in breast treatments.

**Figure 2:**
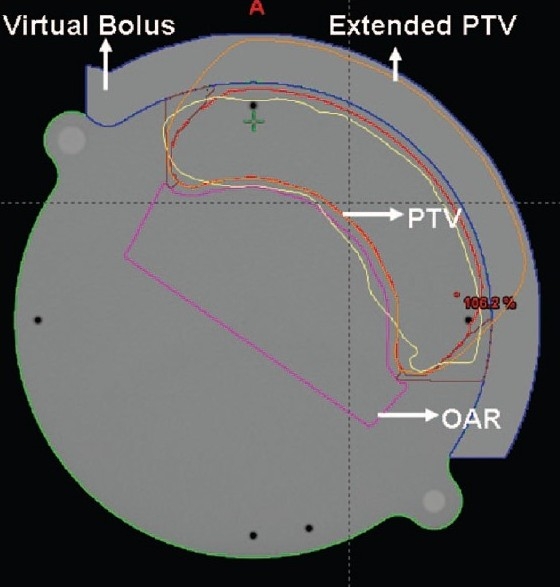
Extended PTV and artificial bolus defined in the data set to create the intensity extension outside skin region

In our study we used a field extension of two cm outside the phantom surface to simulate skin flashing that is normally used in patient treatment. To create an automatic intensity extension in virtual bolus IMRT planning, a new PTV was created by extending the target volume two cm outside the skin [Figure 2]. This extended PTV was used in IMRT optimization to prescribe the dose along with the original target volume.

### Treatment Plans

Many treatment planning approaches have been suggested and practiced to improve the dose homogeneity in breast radiotherapy.[11–12] For breast-only treatments the quality of the treatment plan can be improved either using forward or inverse IMRT techniques using tangential pair fields. But in situations where the target volume includes breast, supraclavicular and axilla node regions the shape of the target volume becomes highly complex. In this case tangential pair fields alone cannot yield good dose coverage to the whole volume efficiently. Under these circumstances conventionally beam edge matched asymmetric tangential pair arrangement for breast region and AP-PA pair for supraclavicular region is in use. In volume based IMRT method this entire volume can efficiently be covered with multiple fields.[13]

To study the effect of different planning techniques on skin dose we have created the following treatment plans; tangential wedge pair plan, segmented tangential pair plan, inversely optimized tangential pair IMRT plan and inversely optimized multiple field IMRT plan. Seven treatment fields with different gantry angles ranging from 310° to 160°, in the anterior direction, are used to generate the IMRT plans in multiple field technique. To study the effect of intensity extension on skin dose and PTV dose coverage in IMRT, plans are generated with and without intensity extension in both tangential pair and multiple field technique.

### Intensity Extension

In forward treatment planning techniques such as tangential wedge pair treatment field outside the skin can be extended simply by defining the treatment portal as required. But in volume based optimization techniques the beam intensity is optimized by the inverse treatment planning algorithm to cover the defined target volume based on the dose-volume constraints. Since the treatment planning system defines a zero dose region outside the skin the inverse optimization tends to fail if the target volume is extended beyond the skin region to achieve the intensity extension.. The reason for this failure is already mentioned in the introduction section. To overcome this problem and extend the beam fluence outside the skin region the following methods were used in our study.

**Figure 3:**
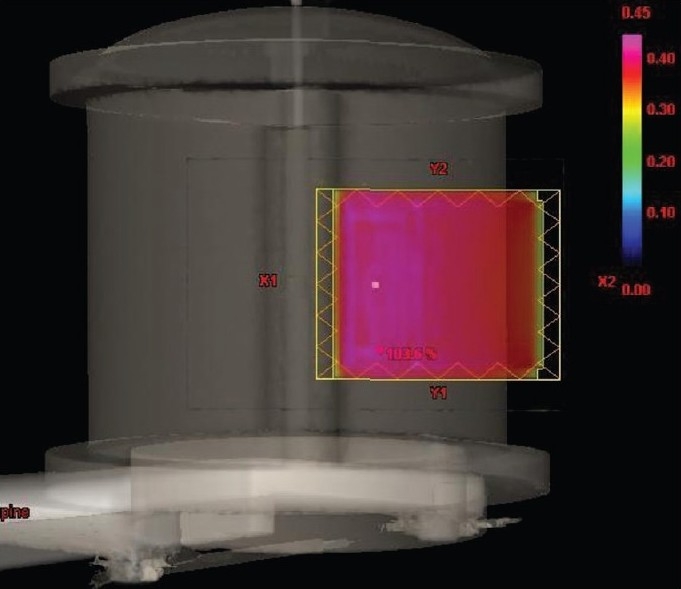
Beam fluence with intensity extension outside skin region

In the first method, IMRT plans are generated using the original target volume. Then the intensity of the optimized beam outside the skin is extended using the skin flash tool available in the planning system. The skin flash tool is a paint brush tool which allows the user to extend the fluence outside the skin in the beam's eye view (BEV). The intensity value for skin flashing is selected using a cut range parameter available in the skin flash tool. Based on the cut range parameter value the skin flash tool will go through the intensity cells inside the field from the edge and extend that intensity value outside. So the cut range parameter should be carefully selected to avoid hot and cold regions in the extended fluence. In our study we used a cut range parameter value of five mm to extend the fluence uniformly two cm outside the skin. The beam fluence with intensity extension outside the skin region of a tangential pair IMRT field is shown in Figure 3.

In the second method the PTV expanded outside the skin region was used to obtain the intensity extension. In this method an artificial bolus of thickness two cm is created to encompass the region of PTV outside the skin [Figure 2]. A mean Hounsfield number of 85, obtained from the phantom image was defined to the bolus. In IMRT planning, the optimization was performed by prescribing the dose to the expanded PTV in addition to the original target volume. This results in extended IM beam beyond the skin region. From this optimized beam the deliverable beam and the dose calculations were performed without considering bolus in the dose calculation. Thus the bolus is used only to calculate extended fluence and plays no role in the final MU and dose calculation.

### Dose Measurement

PTW *in vivo* diode detectors have been used in entrance dose measurement. The detectors used were “p-type” semiconductor diodes with an inherent two mm lead build up cap and can be used in the energy range of 5 MV to 13 MV photons. The entrance calibration factors, generated for the individual detectors, were used to convert the meter reading into dose. Since the diode response is more susceptible to angle of beam incidence, field size, Source to Detector Distance (SDD) and dose rate the characteristics of the individual diodes are studied and appropriate correction factors are used in the dose calculation.

The skin dose was measured at six different points as shown in Figure 4a and 4b. The points were chosen so that two were situated at apex and two were on either side of the target volume. The points are named as A, B, C, D, E and F as shown in Figure 4b.

**Figure 4a:**
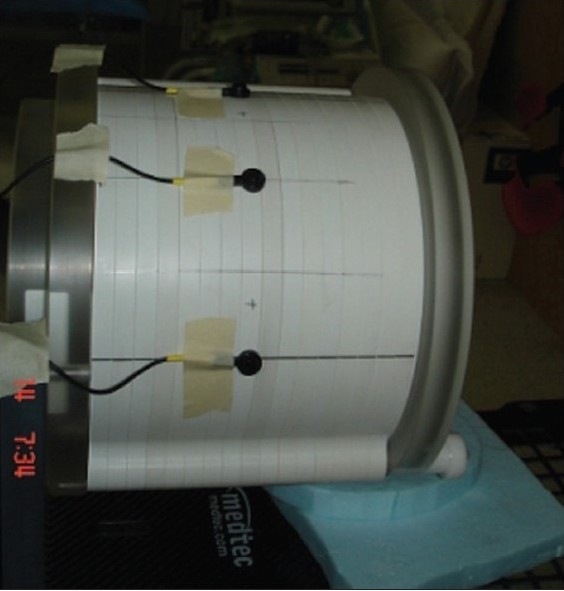
Positions of the diode detectors on the phantom surface for entrance dose measurements

**Figure 4b:**
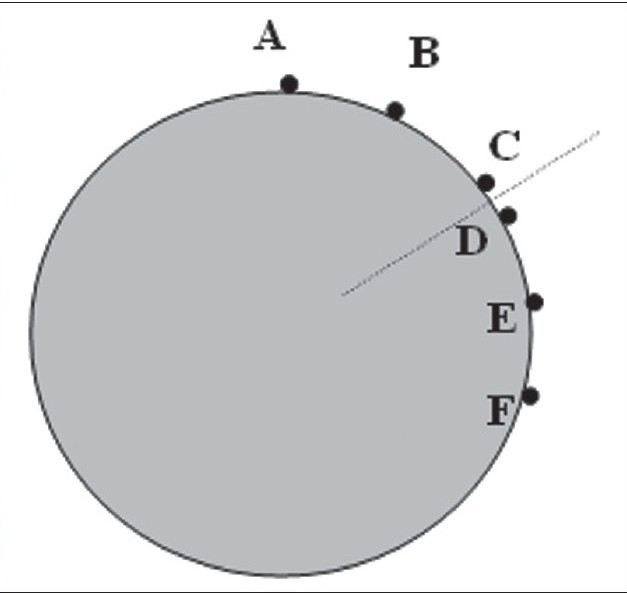
Detailed positions of all measurement positions of detectors in axial plane

### Patient Plans

The impact of intensity extension on the PTV and critical structures' dose was studied by generating the IMRT plans with and without intensity extension on the patient image data set. Plans with intensity extension using skin flash tool and virtual bolus were generated for the dose comparison. Two sets of patient images were used; one with breast-only treatment and another with breast and supraclavicular region. Tangential pair field arrangement was used for the breast-only planning and multiple fields at different gantry angles were used for the breast and supraclavicular planning. The dose delivered to PTV, Lung, Heart and Contra-lateral Breast was compared using corresponding DVHs.

## Results and Discussion

### PTV Dose Volume Histograms

**Figure 5:**
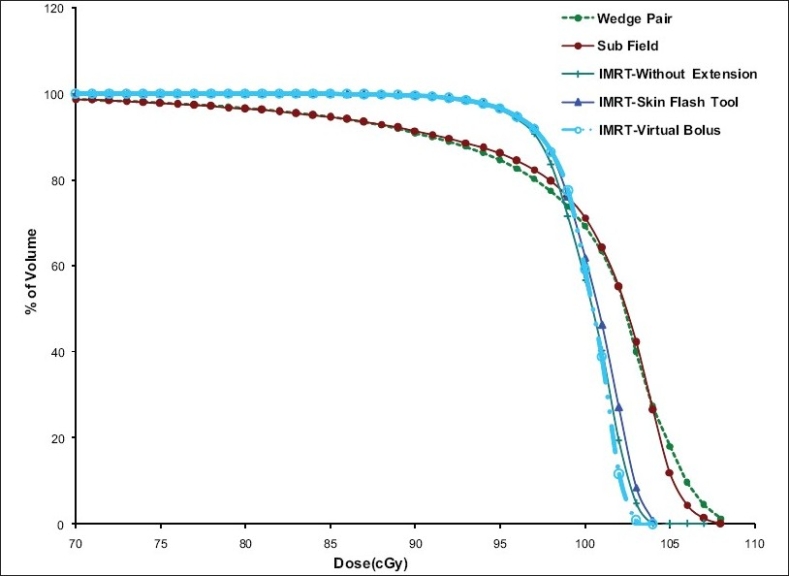
Dose Volume Histograms (DVH) of target volume obtained from different tangential pair plans

**Figure 6:**
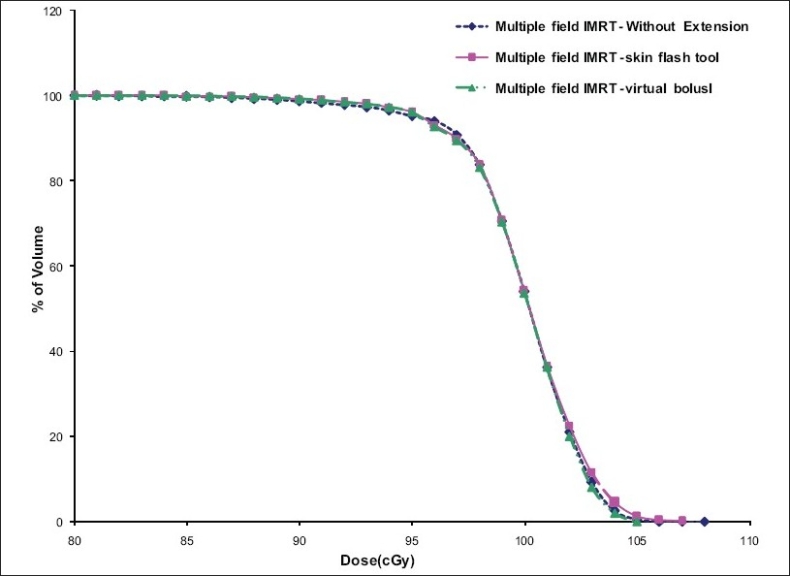
DVHs of target volume obtained from different multiple beam IMRT plans

PTV DVHs that resulted from tangential pair plans and multiple field IMRT plans are shown in figure 5 and 6 respectively. Generally the PTV dose coverage was better in IMRT plans compared to the tangential wedge pair and segmented beam plans. The volume receiving high dose was significantly less in IMRT plans compared to tangential wedge pair and segmented beam plans. The PTV dose coverage and the dose homogeneity are marginally better in the virtual bolus method compared to plans without extension and skin flash tool method [Figure 5]. The reason for this is, in the virtual bolus method the optimum intensity is decided by the iterative process in the extension region, whereas in the skin flash tool method the extension is taken from five mm inside the field edge, which could result in high dose region near the surface. Multiple beam IMRT plans also show a similar trend [Figure 6] but the magnitude of difference is insignificant.

### Entrance Dose

Since diodes show significantly different characteristics in entrance and exit measurements, only the entrance dose that resulted from each beam at the measurement point is used for the analysis to avoid the ambiguity.[14] Table 1 shows the entrance dose measured at different positions on the phantom [figure 4b] for tangential pair plans. Positions A, B and C were used to measure the entrance dose from Field 1 and positions D, E and F were used to measure the dose from field 2 of the tangential pair plans. Since the positions C and D are close to apex region of PTV the measured dose values are close to half of the prescription dose (50%).Since the points B, E and A, F are away from the apex region and at the gradually increasing broadened region of the target volume the measured doses also gradually increase. This is either due to toe end of the wedge in conventional plan or the increased beam fluence in IMRT fields. From the dose values at positions C and D of the IMRT plans it is clear that there is no significant difference in entrance dose at the apex region of the target due to different methods used for intensity extension. The entrance dose values of position C and D in the IMRT plan without intensity extension are significantly low (only ∼10% of the dose with extension). This is because the diode detectors have a three mm base material below the active region of measurement. Hence, the point of measurement becomes three mm above the surface of the phantom. Since the fields do not have an extension beyond the phantom surface in the IMRT plan without extension the measured dose is significantly lower. This gives an idea about the potential under dosage in the surface region due to small positional errors or patient movement in IMRT plans without intensity extension.

**Table 1:** Entrance Dose Measurements at different positions in Tangential Pair Treatment Plans

*Treatment Plan*	*Dose (cGy)*					
	
	*A*	*B*	*C*	*D*	*E*	*F*
Wedge Pair	73.85	69.31	52.30	52.16	68.24	73.40
Sub Field Technique	73.52	69.78	54.20	53.80	67.30	73.39
IMRT without Extension	70.80	67.84	5.52	5.61	65.85	70.52
IMRT –Skin Flash Tool Extension	71.04	67.98	51.85	51.90	65.70	70.11
IMRT –Virtual Bolus Method	70.40	67.66	51.02	51.07	65.75	70.24

Table 2 shows the entrance dose that resulted from different fields at different positions in multiple field IMRT plans. Depending on the beamlet weight and number of beams entering through the measurement point, different positions receive different dose. There is no significant difference in measured entrance dose due to different intensity extension methods used in planning [Table 2]. Since points A, B, C, D and E are in the skin flash region of the fields the dose values are less in the IMRT plan without extension compared to the plan with extension and the magnitude of difference is high in the apex position of the target (C and D). Since the measurement position F does not require the intensity extension from any of the fields its value is same in all multiple field IMRT plans.

**Table 2:** Entrance Dose Measurements at different positions in Multiple Field IMRT Plans

*Treatment Plan*	*Dose (cGy)*					
	
	*A*	*B*	*C*	*D*	*E*	*F*
IMRT without extension	60.72	86.72	57.57	57.36	83.78	66.07
IMRT –Skin Flash Tool Extension	68.38	94.80	95.27	93.04	92.66	67.24
IMRT –Virtual Bolus Method	68.64	94.52	94.26	93.24	92.48	66.94

### Patient Plan comparison

**Figure 7:**
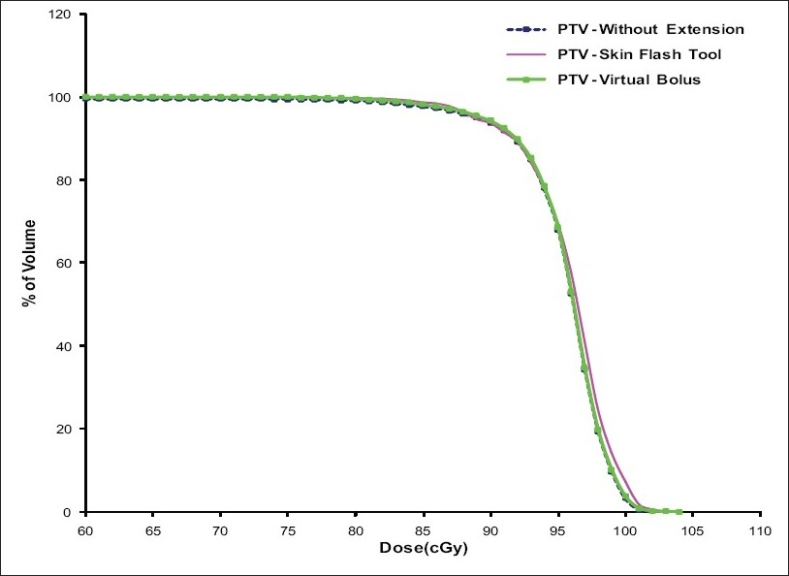
Comparison of PTV – DVH resulted from tangential fields IMRT plans with and without intensity extension

**Figure 8:**
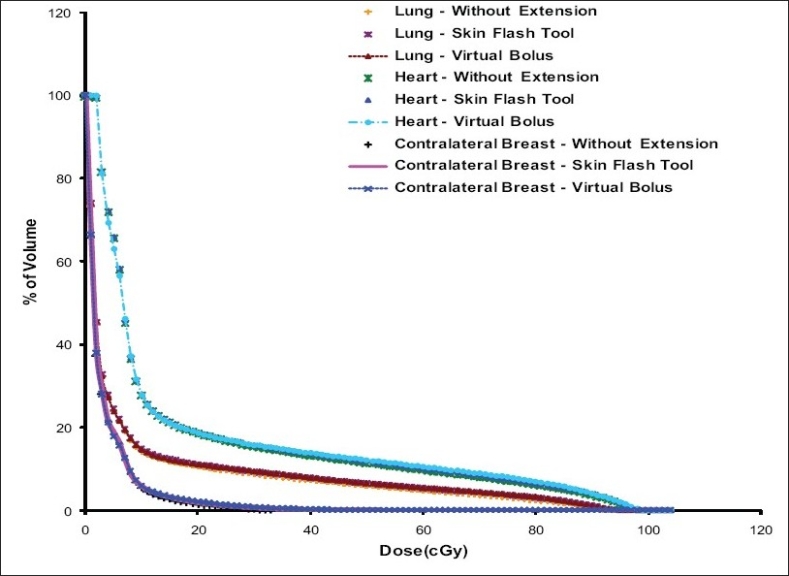
Comparison of Lung, Heart and Contra-lateral Breast DVHs resulted from tangential fields IMRT plans with and without intensity extension

**Figure 9:**
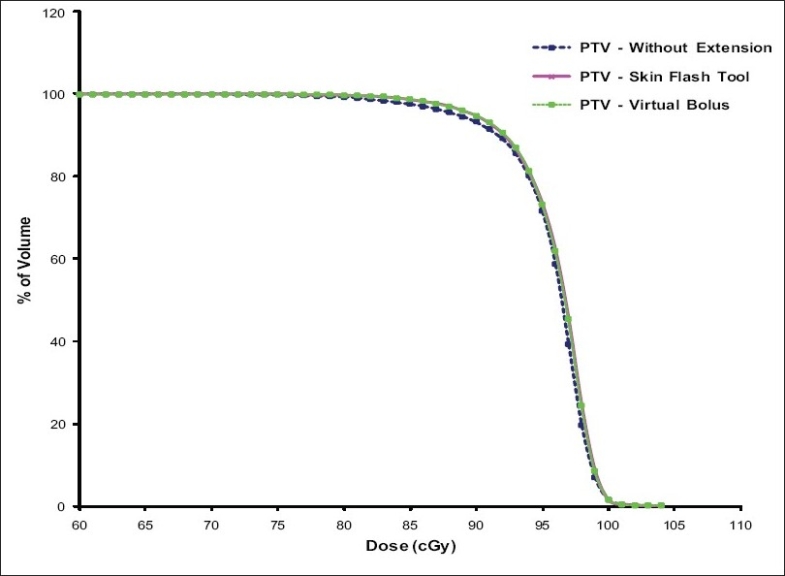
Comparison of PTV – DVH resulted from multiple field IMRT plans with and without intensity extension

**Figure 10:**
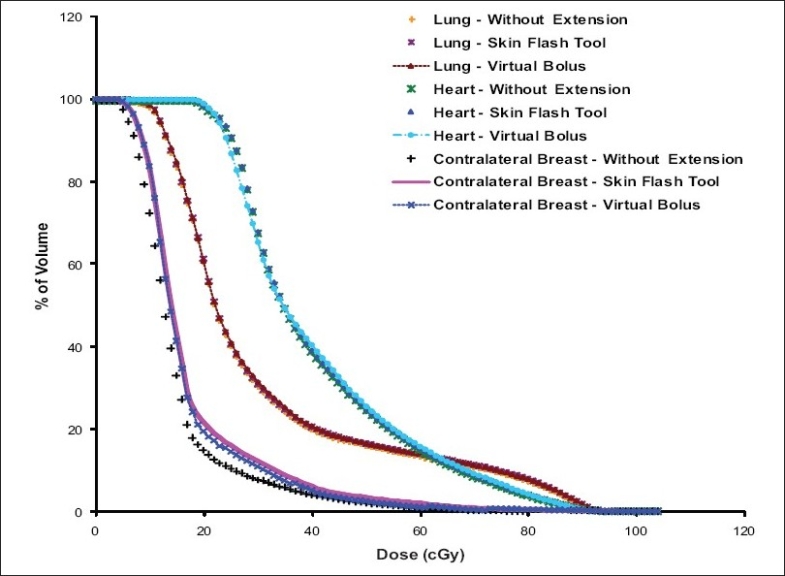
Comparison of Lung, Heart and Contra-lateral Breast DVHs resulted from multiple field IMRT plans with and without intensity extension

Figure 7 shows the PTV-DVH that resulted from tangential pair IMRT plan with and without intensity extension. The PTV dose coverage is almost same in all plans except a small volume of PTV receives higher dose in plan with skin flash tool based intensity extension compared to other plans. The reason for this is already discussed in the section *“PTV Dose Volume Histograms”*. Figure 8 shows the dose received by critical structures like Lung, Heart and Contra-lateral breast in tangential pair IMRT plans. As can be seen from the figure, there is no significant difference in dose received by these structures in different plans. Figures 9 and 10 shows the PTV and critical structures DVH that resulted from multiple field IMRT plan with and without intensity extension. There is no significant difference in dose received by PTV, lung and heart in different plans. But the dose received by contra-lateral breast is more in plan with intensity extension compared to plan without extension [figure 10]. This is because some of the beam angles exit through the contra-lateral breast in multiple fields IMRT plans. Extending the field intensity beyond skin region to those fields results in irradiating more volume of the contra-lateral breast and in more dose delivery.

## Conclusions

For the defined target volume IMRT plans give superior dose coverage and dose homogeneity compared to the conventional plans. Intensity extension beyond the skin region must be incorporated in volume based optimization techniques to account for intrafraction motion and setup errors. In multiple field IMRT plans incorporation of intensity extension increases the contra-lateral breast dose. There is no significant difference in entrance dose, especially in the apex region of the target volume, due to different methods of intensity extension used in the IMRT planning. Care should be taken to avoid hot and cold regions, in the extended intensity region, while selecting skin flash tool parameters,.
